# Personal distress and the influence of bystanders on responding to an emergency

**DOI:** 10.3758/s13415-016-0423-6

**Published:** 2016-04-28

**Authors:** Ruud Hortensius, Dennis J. L. G. Schutter, Beatrice de Gelder

**Affiliations:** Brain and Emotion Laboratory, Department of Cognitive Neuroscience, Faculty of Psychology and Neuroscience, Maastricht University, Maastricht, The Netherlands; Cognitive and Affective Neuroscience Laboratory, Department of Medical and Clinical Psychology, Tilburg School of Social and Behavioral Sciences, Tilburg University, Tilburg, The Netherlands; Department of Psychiatry and Mental Health, Faculty of Health Sciences, University of Cape Town, Cape Town, South Africa; Donders Institute of Brain, Cognition and Behaviour, Radboud University Nijmegen, Nijmegen, The Netherlands

**Keywords:** Personal distress, Sympathy, Bystander effect, Action preparation, Helping behavior, Motor corticospinal excitability

## Abstract

Spontaneous helping behavior during an emergency is influenced by the personality of the onlooker and by social situational factors such as the presence of bystanders. Here, we sought to determine the influences of sympathy, an other-oriented response, and personal distress, a self-oriented response, on the effect of bystanders during an emergency. In four experiments, we investigated whether trait levels of sympathy and personal distress predicted responses to an emergency in the presence of bystanders by using behavioral measures and single-pulse transcranial magnetic stimulation. Sympathy and personal distress were expected to be associated with faster responses to an emergency without bystanders present, but only personal distress would predict slower responses to an emergency with bystanders present. The results of a cued reaction time task showed that people who reported higher levels of personal distress and sympathy responded faster to an emergency without bystanders (Exp. 1). In contrast to our predictions, perspective taking but not personal distress was associated with slower reaction times as the number of bystanders increased during an emergency (Exp. 2). However, the decrease in motor corticospinal excitability, a direct physiological measure of action preparation, with the increase in the number of bystanders was solely predicted by personal distress (Exp. 3). Incorporating cognitive load manipulations during the observation of an emergency suggested that personal distress is linked to an effect of bystanders on reflexive responding to an emergency (Exp. 4). Taken together, these results indicate that the presence of bystanders during an emergency reduces action preparation in people with a disposition to experience personal distress.

**Electronic supplementary material:**

The online version of this article (doi:10.3758/s13415-016-0423-6) contains supplementary material, which is available to authorized users.

When confronted with a person in distress, most people react to the situation by rushing forward to help. Generally, two types of emotional reactions to an emergency situation that promote helping behavior are distinguished—namely, personal distress and sympathy (for a review, see Batson, Fultz, & Schoenrade, 1987). Both state and trait levels of sympathy and personal distress have been linked to helping behavior (Archer, Diaz-Loving, Gollwitzer, Davis, & Foushee, 1981; Carlo et al. 1999; Cialdini et al., 1987; Coke, Batson, & McDavis, 1978; Eisenberg, Fabes, et al., 1989; Eisenberg & Miller, 1987; Eisenberg, Miller, et al., 1989). However, sympathy and personal distress markedly differ in terms of their underlying motivations. The former results in altruistic-driven (other-oriented; i.e., feelings of sympathy and compassion for the victim) and the latter in egoistic-driven (self-oriented; i.e., feelings of distress and discomfort in the onlooker) helping behavior (Batson et al., 1987; Batson, O’Quin, Fultz, Vanderplas, & Isen, 1983; Davis, 1983).

Although feelings of personal distress and sympathy each lead to helping behavior, the underlying incentives to help may thus be very different. As such, it could be argued that social situation or context could have different influences on these two factors. Indeed, studies have shown that helping behavior driven by personal distress is reduced when the aversive situation can be easily avoided, whereas sympathy-driven helping behavior is not (Batson, Duncan, Ackerman, Buckley, & Birch, 1981; Batson et al. 1983; Coke et al., 1978; Toi & Batson, 1982). Contextual effects have also been reported for trait measures of other- and self-oriented responses to emergency situations (Batson, Bolen, Cross, & Neuringer-Benefiel, 1986; Carlo, Eisenberg, Troyer, Switzer, & Speer, 1991; Romer, Gruder, & Lizzadro, 1986). Romer and colleagues (1986) reported that people with an altruistic orientation offered the most help when no compensation (experimental credits) was given. Interestingly, helping was reduced in this group when compensation was offered. Furthermore, social evaluation of the latent helper by the experimenter influences the relation between personal distress and helping behavior, but not between sympathy and helping behavior (Archer et al., 1981; Eisenberg, Miller, et al., 1989; Fultz, Batson, Fortenbach, McCarthy, & Varney, 1986). For example, directly manipulated and self-reported concerns for social evaluation did not account for the positive relation between trait sympathy and helping behavior (Eisenberg, Miller, et al., 1989). In sum, social context has a more pronounced and negative influence on the relation between personal distress and helping behavior than on helping behavior driven by sympathy.

Helping behavior also decreases when more people are present at the scene. This phenomenon is known as the *bystander effect* (Darley & Latané, 1968). Several cognition-based explanations, including notions like the diffusion of responsibility or pluralistic ignorance have been given for this lack of helping behavior (Latané & Darley, 1970). The decision model proposed by Latané and Darley (1970) describes the explicit cognitive calculation in terms of attentional capture, evaluation, responsibility, beliefs, and the conscious decision to help. Interference can occur at any of these levels. However, this model does not cover the entire range of explanations (see, e.g., Garcia, Weaver, Moskowitz, & Darley, 2002) or the emerging view on helping behavior and prosocial behavior (Preston, 2013; Preston & de Waal, 2002; Rand & Nowak, 2013; Zaki, 2014). Helping behavior is observed across species, ranging from rats (Ben-Ami Bartal, Decety, & Mason, 2011; Ben-Ami Bartal, Rodgers, Bernardez Sarria, Decety, & Mason, 2014; Márquez, Rennie, Costa, & Moita, 2015; Sato, Tan, Tate, & Okada, 2015) to chimpanzees (Warneken & Tomasello, 2006; Warneken, Hare, Melis, Hanus, & Tomasello, 2007). The act of helping is not necessarily a deliberate one. As was described by Preston (2013), providing help is rooted in an evolutionarily conserved mechanism, offspring care, with fixed action patterns (see also Decety, Norman, Berntson, & Cacioppo, 2012). This bottom-up view highlights the importance of a neural mechanism for fast, context-dependent, goal-directed responses. Merely the observation of a salient situation—for example, witnessing a person in distress—triggers a wide variety of reflexive responses (Preston & de Waal, 2002), including increased action readiness and preparation (e.g., fight–flight responses) (de Gelder, Snyder, Greve, Gerard, & Hadjikhani, 2004; Frijda, 1986; Grèzes & Dezecache, 2014; Hajcak et al., 2007; Lang, Greenwald, Bradley, & Hamm, 1993; Schutter, Hofman, & van Honk, 2008).

In a recent functional magnetic resonance imaging (fMRI) study, we investigated the neural basis of the bystander effect by manipulating the number of bystanders present at an emergency (Hortensius & de Gelder, 2014). The results showed that activity decreased in the left precentral and postcentral gyrus and medial frontal gyrus with an increase in the number of bystanders when participants witnessed an emergency. This suggests that the number of bystanders influences neural responses in brain regions dedicated to motor-related behavior, possibly indicative of action preparation during the observation of an emergency (de Gelder et al., 2004; Hajcak et al., 2007; Pichon et al. 2012; Schutter, Hofman, et al. 2008). An outstanding question is how trait levels of sympathy and personal distress influence the effect of bystanders on action preparation.

In the present study, we examined the extent to which trait sympathy and personal distress predicted reaction times during a cued reaction time task when participants witnessed an emergency without bystanders (Exp. 1), and when the number of bystanders was manipulated during an emergency (Exp. 2). We hypothesized that both trait personal distress and sympathy would predict faster responses to an emergency, as compared with a nonemergency situation without bystanders (Exp. 1). Furthermore, we expected that an increase in the number of bystanders would result in slower responses to an emergency situation. On the basis of the previously found negative influence of social context (e.g., possibility to escape the situation or exposure to social evaluation) on helping behavior driven by personal distress, we anticipated that this slowing of reaction times with an increase in the number of bystanders would be predicted by personal distress and not by sympathy (Exp. 2).

## Experiments 1 and 2

### Method

#### Participants

Sixty-two volunteers, between 18 and 29 years of age, participated in exchange for course credits. In all, 18 female and 12 male students took part in Experiment 1, and 21 different female and 11 different male students took part in Experiment 2. Right-handed (*n* = 56), left-handed (*n* = 5), and ambidextrous (*n* = 1) participants were included. In Experiment 2, the data from two participants were lost due to technical failure and were replaced by new volunteers. Participants were unaware of the aim of the study. Written informed consent was obtained, and the experiment was carried out in accordance with the standards set by the Declaration of Helsinki.

#### Stimuli

**Fig. 1:**
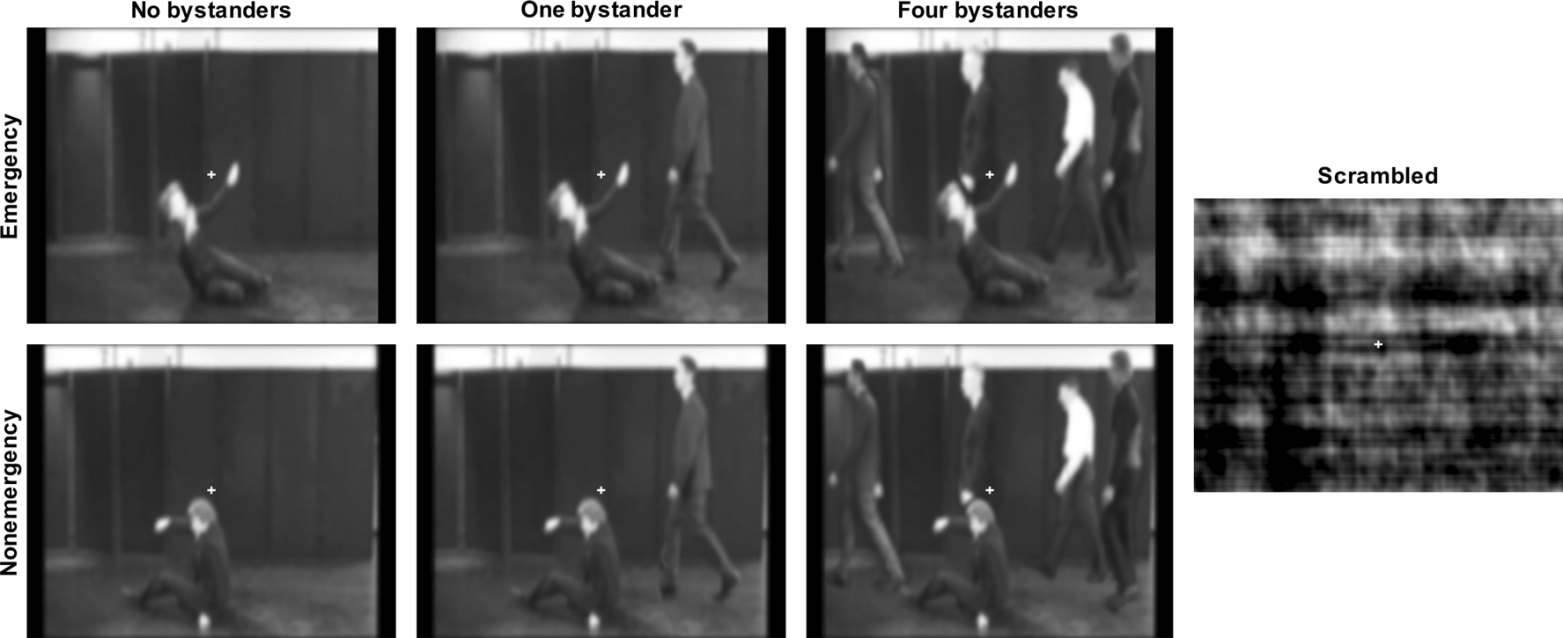
Stills of the stimuli used in the study

The stimuli used in Hortensius and de Gelder (2014) were slightly modified for the present purpose. A simulation of a street-side event was used as a starting point to create an emergency situation in which a woman was shown fainting and falling to the floor. During this emergency, people were passing by (henceforth, the bystanders). The original short video clips were recorded from the viewpoint of a person looking across the street. The grayscale video clips were blurred in order to reduce the visibility of facial expressions and other nonrelevant information. In the present experiment, we made several changes to the existing video clips. First and foremost, besides the fainting and falling scenario, we created a nonemergency situation in which the woman stood up in a completely natural way. Using Photoshop CS2 (Adobe Systems Inc., San Jose, CA, USA) we overlaid the bystander sequence on both types of situations. This ensured that (a) the emergency and nonemergency situations were similar in terms of bystanders, but differed only in the action of the woman, and (b) the actions of the woman were similar for all of the bystander conditions. Both the increase in visual complexity between the emergency and nonemergency situations and the emotional impact of the woman’s actions with different numbers of bystanders were kept similar. To increase realism, actions of the target character and bystanders happened within the same time window. In total, six scenarios were created, with the situation being either an emergency (woman falling) or a nonemergency (woman standing up) and the number of bystanders consisting of none, one, or four bystanders. Six unique videos per scenario (three different actors and groups, with two repetitions) lasting 1 s were created. See Fig. 1 and the supplemental online materials for examples of the stimuli used.

In addition, scrambled versions of the videos were made in MATLAB (version R2011b; The MathWorks Inc., Natick, MA, USA) using a Fourier-based transformation of each phase spectrum of every video frame. This procedure removes all social–emotional information except for low-level visual parameters such as movement and spatial frequency. These scrambled videos served as a low-level visual control condition.

#### Task

**Fig. 2:**
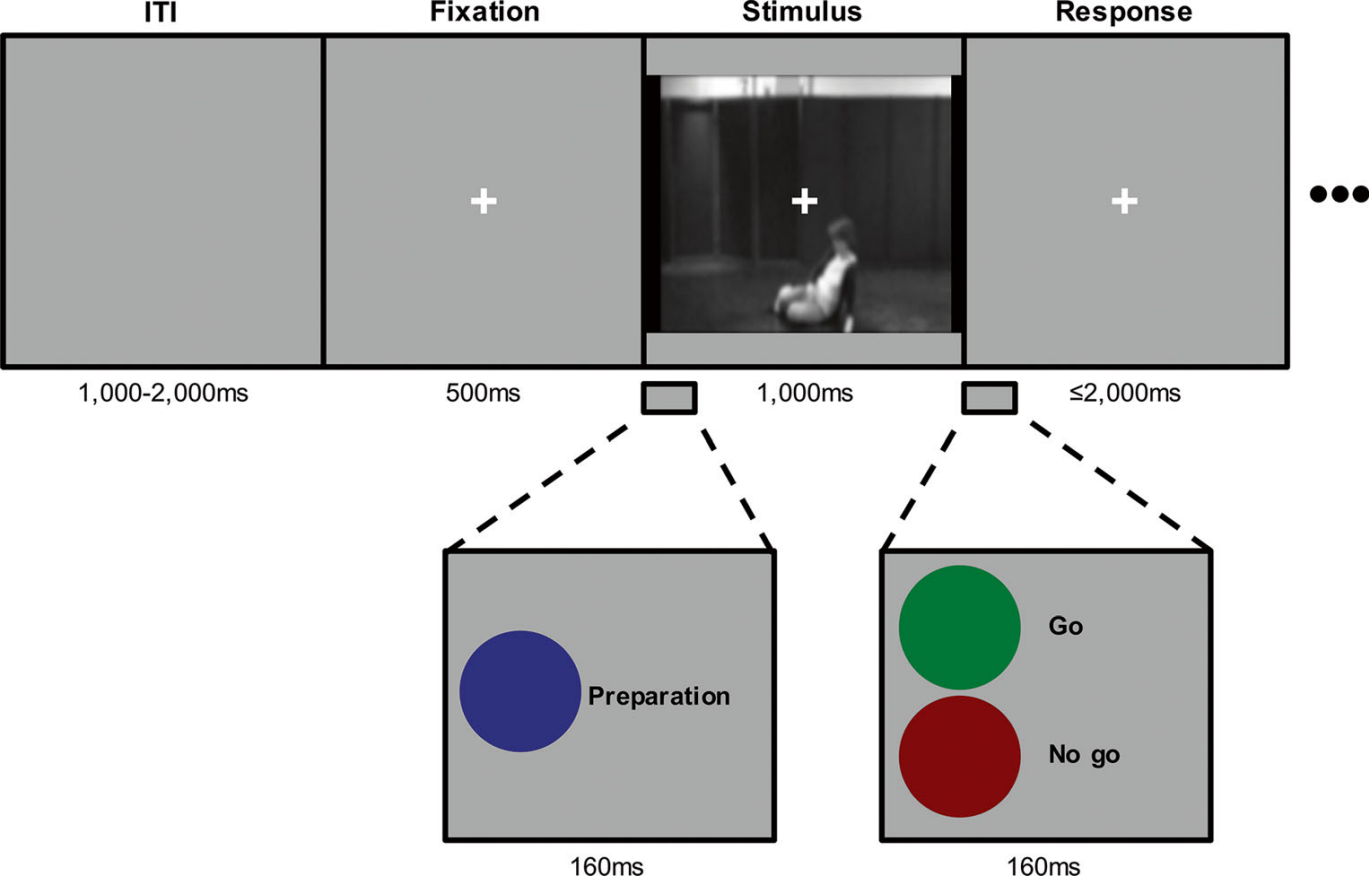
Cued reaction time task. Between a preparation cue and response cue, a video clip was shown. Participants responded as quickly as possible to the go cue with the index finger of their dominant hand. ITI, intertrial interval

To measure the effect of an emergency on reaction times, an adapted cued reaction time task was used. In a cued reaction time task, a preparation cue is presented before a response cue, which allows the participants to prepare their response (Hagura, Kanai, Orgs, & Haggard, 2012; van Boxtel & Böcker, 2012). A video clip was presented in between the onsets of the preparation cue and the go cue (Fig. 2). Following a preparation cue (blue dot) at the onset of the video clip, a go cue (green dot) was presented after 1 s—that is, the offset of the video clip—to inform the participant to respond. Both cues were presented for 160 ms. On 20 % of the trials in Experiment 1, and 12.5 % of the trials in Experiment 2, a no-go cue (red dot) was presented.

Importantly, the social situation might influence ongoing emotional and cognitive processes that could be measured by means of reaction times after the offset of the video. This rationale was taken from other studies using emotional Stroop tasks, gaze-cueing paradigms, and emotional go/no-go tasks to map individual differences in processing emotional and social information (Mathews & MacLeod, 1994; Nosek, Hawkins, & Frazier, 2011). The present setup allowed us to assess responses to an emergency without explicit measures in a relatively well-controlled environment, and reaction times were taken as an index of action preparation during the nonintentional observation of an emergency.

#### Questionnaire

Empathy can be defined as a multifaceted concept consisting of phenomena including mimicry, sympathy, and perspective taking (Preston & de Waal, 2002). In line with this multidimensional approach, we used the interpersonal reactivity index (IRI) to measure trait empathy (Davis, 1980, 1983; De Corte et al., 2007). This questionnaire assesses several different aspects of empathy. Besides perspective taking and fantasy (i.e., the ability to transpose oneself to a fictional situation), the IRI measures empathic concern and personal distress. The former trait measures sympathy and compassion for less fortunate others (i.e., an other-oriented emotional reaction), whereas the latter measures the experience of discomfort in response to distress in others (i.e., a self-oriented emotional reaction). The difference between personal distress and empathic concern becomes clear when one looks at some of the example items to measure personal distress—for instance, “I tend to lose control during emergencies” and “When I see someone who badly needs help in an emergency, I go to pieces”—and empathic concern—for instance, “I often have tender, concerned feelings for people less fortunate than me” and “I am often quite touched by things that I see happen.” In the literature, a variety of terms are used to describe an other-oriented emotional response to the distress of another person (Batson, 2009). *Empathic concern* and *sympathy*, the most commonly used labels in the literature, are often used interchangeably. The use of the term *empathic concern* might, however, result in confusion, because it suggests that empathic concern and empathy are the same. *Empathy* refers to the multifaceted concept, whereas *empathic concern* is an aspect of this concept (Wispé, 1986). In line with the existing literature, we will use the term *sympathy* when referring to the trait measure of an other-oriented emotional reaction.

#### Procedure

The task and experimental procedure were identical for both experiments, unless otherwise specified. The experimental session started with six practice trials of the cued reaction time task, using video clips of a woman standing and waiting. Next, participants completed a baseline block with the scrambled versions of videos used in the subsequent experimental blocks. In Experiment 1, only the two scenarios without bystanders were used in the main experimental blocks, whereas in Experiment 2, all six scenarios were used. No mention was made with respect to the content of the movies. Original and mirrored videos were included to prevent a possible influence of the direction of movement in the videos (e.g., left motion direction) on the subsequent response. The stimuli were presented in a randomized order and repeated twice, resulting in 24 go trials per condition. Participants were instructed to respond as quickly as possible with the index finger of their dominant hand and to fixate on the fixation cross shown continuously during the task. After the cued reaction time task, participants completed the Dutch version of the IRI.

#### Data reduction and analysis

Reaction times <150 ms or >1,500 ms were removed from analysis (mean ± *SD* percentages of trials removed: Exp. 1, 5.33 ± 1.43 %; Exp. 2, 3.12 ± 2.18 %), as well as incorrect trials (mean ± *SD* percentages of false alarms and misses: Exp. 1, 2.30 ± 2.48 %; Exp. 2, 2.27 ± 2.17 %). Reaction times were calculated as percentage changes from the baseline (scrambled) block (set at 100 %).

In Experiment 1, a paired-sample *t* test was performed to look at the difference in reaction times between emergency and nonemergency situations. In addition, we subtracted the baseline-corrected reaction times of the nonemergency from those of the emergency trials to calculate a bias score, so that negative values indicated faster responses to the emergency situation. In Experiment 2, a general linear model (GLM) for repeated measurements with Situation (two levels) and Number of Bystanders (three levels) as within-subjects factors was used to test the difference in the influences of the number of bystanders on baseline-corrected reaction times during the observation of an emergency versus a nonemergency situation. Given the a priori predictions, we tested for a significant linear trend contrast. Paired-samples *t* tests were used for the post-hoc tests.

To investigate the relationship between trait personal distress and sympathy and responses to an emergency, linear regression analyses were employed for both experiments. In the first step, the hypothesized predictors were entered into the model (for Exp. 1, trait personal distress and sympathy; for Exp. 2, trait personal distress), whereas in Step 2, the remaining scales were added in a stepwise fashion (method: probability of *F* to enter, <.05; criteria probability of *F* to remove, >.1). Cohen’s effect size (ƒ^2^) was calculated using the formula ƒ^2^ = *R*^2^/(1 – *R*^2^), with effect sizes of around 0.02, 0.15, and 0.35 being interpreted as small, medium, and large, respectively (Cohen, 1988). The alpha level of significance was set at .05 (two-tailed).

## Results

### Experiment 1

**Fig. 3:**
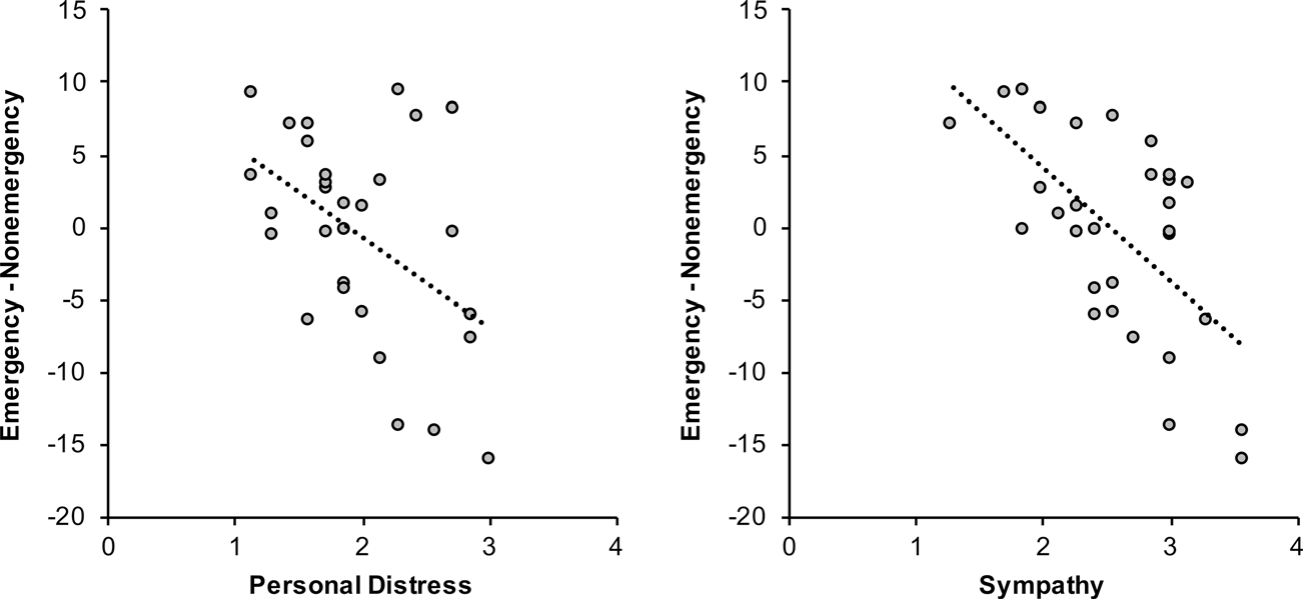
Trait personal distress and sympathy predicted faster responses to an emergency than to a nonemergency situation without bystanders

No difference in reaction times was found between emergency (mean ± *SEM* percentage change from baseline: 98.56 ± 1.19 %) and nonemergency (99.13 ± 1.18 %) situations, *t*(29) = −0.44, *p* = .66. A significant linear regression model was observed for the emergency–nonemergency bias score, *F*(2, 27) = 13.16, *p* < .001, *R*^2^ = .49, ƒ^2^ = 0.96 (Table 1). In line with our expectations, participants with higher self-reported trait personal distress, β = −.35, *p* = .02, and sympathy, β = −.54, *p =* .001, responded faster to an emergency than to a nonemergency situation without bystanders (Fig. 3).

**Table 1:** Outcome of the regression analysis for emergency – nonemergency bias scores in Experiment 1

	*b*	β	*p*
*Step 1*			
Overall model: *F*(2, 27) = 13.16, *p* < .001, *R* ^2^ = .49, ƒ^2^ = 0.96			
Constant	29.36 ± 5.95 [17.15, 41.57]		<.001
Personal distress	−0.67 ± 0.27 [−1.22, −0.12]	–.35	.02
Sympathy	−0.98 ± 0.25 [−1.50, −0.46]	–.54	.001
*Step 2*			
Perspective taking^*^		.16	.29
Fantasy^*^		.09	.58

*b* = unstandardized coefficients ± standard errors [95 % confidence intervals], β = standardized coefficient. ^*^Removed predictors

### Experiment 2

Table 2 shows the reaction times across conditions. No main effect of situation, *F*(1, 29) = 3.69, *p =* .07, or number of bystanders, *F*(2, 58) = 0.40, *p =* .67, was observed. Contrary to expectations, no significant interaction between situation and number of bystanders was found, *F*(2, 58) = 1.43, *p =* .25. The linear trend for this interaction was also not significant, *F*(1, 29) = 0.99, *p =* .33, indicating that the linear effect of the number of bystanders on reaction times did not vary as a function of the situation. To determine whether trait personal distress predicted the effect of an increase in the number of bystanders during an emergency situation, we calculated the regression slope of the reaction times as a function of the number of bystanders in each situation (emergency and nonemergency). This analysis was adapted from the perceptual-processing literature, in which the slope of reaction times is calculated as a function of set size (e.g., Golan, Bentin, DeGutis, Robertson, & Harel, 2014; Lockhart et al., 2014; Wolfe & Horowitz, 2004). In the present study, the slope indicated the change in reaction times with an increasing number of bystanders in line with the previously used parametric approach on group influences (Hortensius & de Gelder, 2014), as well as the finding that the bystander effect grows larger as the number of bystanders increases (Fischer et al., 2011). If the number of bystanders had a disruptive effect on the perception of and reaction to an emergency, people would be slower when the number of bystanders increased, and thus reaction times would increase (positive slope). If, on the other hand, an increase in the number of bystanders had no effect, reaction times would not increase, and the slope would be zero. Finally, a negative slope would indicate a decrease in reaction times: People would respond faster when the number of bystanders increased. Positive or negative slopes might be indicative of a decreased or increased tendency for helping behavior, respectively. The present findings showed a positive slope in both the emergency situation, mean ± *SEM*, 0.48 ± 0.21, and the nonemergency situation, 0.17 ± 0.25.^1^ These slopes were not significantly different from each other, *t*(28) = 1.17, *p* = .25. Although the nonemergency slope did not differ from zero, *t*(28) = 0.67, *p* = .51, however, the emergency slope was significantly larger than zero, *t*(28) = 2.33, *p* = .03.

**Table 2:** Mean reaction times ± standard errors as percentage changes from baseline for Experiment 2

	No Bystanders	One Bystander	Four Bystanders
Emergency	86.34 ± 2.14	87.83 ± 2.13	87.86 ± 1.91
Nonemergency	89.21 ± 1.95	88.24 ± 1.80	89.34 ± 1.96

A significant model was found for the emergency slope, *F*(2, 26) = 4.18, *p =* .03, *R*^2^ = .24,ƒ^2^ = 0.32 (Table 3). Although personal distress was positively associated with the slowing of reaction times with an increase in the number of bystanders, β = .29, *p* = .11, it was neither a significant nor the sole predictor in the model. The slope was predicted by perspective taking, β = .46, *p* = .01.^2^ Interestingly, when responding to the emergency situation, people with higher levels of trait perspective taking showed a stronger effect of the number of bystanders. In other words, people with a disposition to adopt the perspectives of other people became slower when the number of bystanders increased during an emergency. In line with expectations, sympathy did not predict the influence of the number of bystanders, β = .11, *p* = .64. No significant model was found for the nonemergency slope, *F*(1, 28) = 0.59, *p* = .45, *R*^2^ = .02.

**Table 3:** Outcome of the regression analysis of emergency slopes in Experiment 2

	*b*	β	*p*
*Step 1*			
Overall model: *F*(1, 27) = 1.19, *p* = .29, *R* ^2^ = .04			
Constant	−0.19 ± 0.65 [−1.52, 1.15]		.78
Personal distress	0.34 ± 0.31[−0.30, 0.98]	.21	.29
*Step 2*			
Overall model: *F*(2, 26) = 4.18, *p* = .03, *R* ^2^ = .24, ƒ^2^ = 0.32			
Constant	−3.07 ± 1.24 [−5.62, −0.51]		.02
Personal distress	0.48 ± 0.29 [−0.11, 1.07]	.29	.11
Perspective taking	0.96 ± 0.36 [0.21, 1.71]	.46	.01
Sympathy^*^		.11	.64
Fantasy^*^		–.006	.98

*b* = unstandardized coefficients ± standard errors [95 % confidence intervals], β = standardized coefficient. ^*^Removed predictors.

### Discussion

Consistent with our predictions, trait personal distress and sympathy were associated with faster responses to an emergency situation without bystanders present (Exp. 1). However, in contrast to our expectations, personal distress did not significantly predict the effect of the number of bystanders on reaction times during an emergency (Exp. 2). Perspective taking predicted slower responses to an emergency situation with an increase in the number of bystanders. This finding concurs with previous explanations of the bystander effect that appeal to a more cognitive level, including the diffusion of responsibility and pluralistic ignorance (Latané & Darley, 1970). Work by Clark and Word (1972) showed that the bystander effect is driven by ambiguity: Only during ambiguous situations was helping behavior reduced by the presence of bystanders. In the present experiment, the situation could be viewed as more ambiguous when more bystanders were present. This bystander-induced ambiguity may have resulted in an increased need to evaluate the situation, the state of the woman, and the behavior of the bystanders, and especially so for people with higher levels of trait perspective taking. The consequence of this was a slower response to the emergency situation.

Was this slowing in response the result of a slower response selection or increased top-down control of anticipatory responses? In the present cued reaction time task, the preparation cue displayed before a go cue allowed participants to already prepare their response, and this could be influenced by the presented situation (emergency or nonemergency situation). Faster reaction times might thus be indicative of increased action preparation, whereas slower reaction times might indicate a decrease in action preparation. Although in a cued reaction time task like the one used here, reaction times serve as a proxy for action preparation, the difficulty of using reaction times is in distinguishing between the preparation and execution of the response. Moreover, contextual effects on reaction times can be driven by perceptual or action processes, or by a combination of both. One way to overcome this issue would be to directly target the human primary motor cortex by means of transcranial magnetic stimulation (TMS). Using this technique, a noninvasive magnetic pulse is delivered at the surface of the scalp overlying the primary motor cortex. This pulse results in a current flow in the cortex and produces a motor-evoked potential (MEP). Motor corticospinal excitability can be quantified by the MEP amplitude. Single-pulse TMS to map motor corticospinal excitability levels when individuals observe a social cue has been used in studies of action observation (Avenanti et al. 2013; Fadiga, Fogassi, Pavesi, & Rizzolatti, 1995), emotion (Hajcak et al., 2007; Schutter, Hofman, et al. 2008), and empathy (Hétu et al. 2012). Importantly, increases in MEP amplitude has been proposed to index action preparation (Coombes et al., 2009; Hajcak et al., 2007; Schutter, Hofman, & van Honk, 2008; van Loon, van den Wildenberg, van Stegeren, Hajcak, & Ridderinkhof, 2010).

In the third experiment, we used single-pulse TMS to measure changes in motor corticospinal excitability levels, to further substantiate the influence of personal distress. By probing the primary motor cortex of healthy individuals, we aimed to extend the previous experiments by directly quantifying changes in the motor system as a function of the number of bystanders during an emergency situation. TMS studies have shown that when confronted with pain in another individual, both state and trait measures of personal distress increase, rather than decrease, motor corticospinal excitability in the onlooker (Avenanti, Minio-Paluello, Sforza, & Aglioti, 2009). Moreover, trait personal distress has been positively correlated with higher motor corticospinal excitability levels in response to viewing negatively valenced pictures (Borgomaneri, Gazzola, & Avenanti, 2014). These results suggest that a disposition to experience distress can have a direct influence on perception and action. Indeed, a recent study using kinematics showed that trait personal distress predicted reduced motor control in participants who were confronted with another person’s negative emotions (Ferri et al., 2010). In line with these findings, we expected that the linear decrease in motor corticospinal excitability levels as a function of increasing number of bystanders during an emergency would be predicted by personal distress.

Interestingly, previous studies have observed a relationship between trait levels of perspective taking and motor corticospinal excitability levels (Avenanti et al., 2009; Borgomaneri, Gazzola, & Borgomaneri, Gazzola, et al. 2015). These findings were opposite the pattern found for personal distress and were interpreted as stimulation of the (action) state of the observed individual and not as action preparation. Although we did not foresee an effect of perspective taking, these observations suggest that if perspective taking were predictive of the influence of bystanders on responding to an emergency, this effect would be opposite the pattern found for personal distress.

## Experiment 3

### Method

#### Participants

Twenty-three right-handed volunteers (19 women, four men), between 19 and 27 years of age, participated in the experiment in exchange for course credits or payment. Participants were screened for contraindications for noninvasive brain stimulation (Keel, Smith, & Wassermann, 2001). None of the volunteers had a history of psychiatric or neurological disease, and all had normal or corrected-to-normal vision. The participants were naïve to the aim of the study, and written informed consent was obtained. The study was approved by the medical ethics committee of the University Medical Center Utrecht and of Utrecht University, Utrecht, The Netherlands. The stimulation parameters were in agreement with the International Federation of Clinical Neurophysiology safety guidelines (Rossi, Hallett, Rossini, Pascual-Leone, & the Safety of TMS Consensus Group, 2009) and in accordance with the standards set by the Declaration of Helsinki.

#### Transcranial magnetic stimulation

A biphasic magnetic brain stimulator (maximum output 4,160 A peak/1,750 VAC peak) with a modified 8-shaped iron core coil (Neopulse, Atlanta, GA, USA) was used for stimulation over the left M1.

#### Motor-evoked potentials

MEPs were recorded with active Ag–AgCl electrodes (11 × 17 mm) using an ActiveTwo system (BioSemi, Amsterdam, The Netherlands) from the right abductor pollicis brevis (APB) in a belly–tendon montage, with the active electrode placed at the muscle belly of the right APB and the reference electrode located at the proximal phalanx of the thumb (Baumgartner, Willi, & Jäncke, 2007; Hajcak et al. 2007; Schutter, Hofman, et al. 2008). The ground (CMS-DRL) electrode was attached to the wrist. The sampling rate was set at 2048 Hz, and the signal was high-pass filtered offline (3-dB cutoff frequency: 20 Hz, roll-off 24 dB/octave).

#### Procedure

**Fig. 4:**
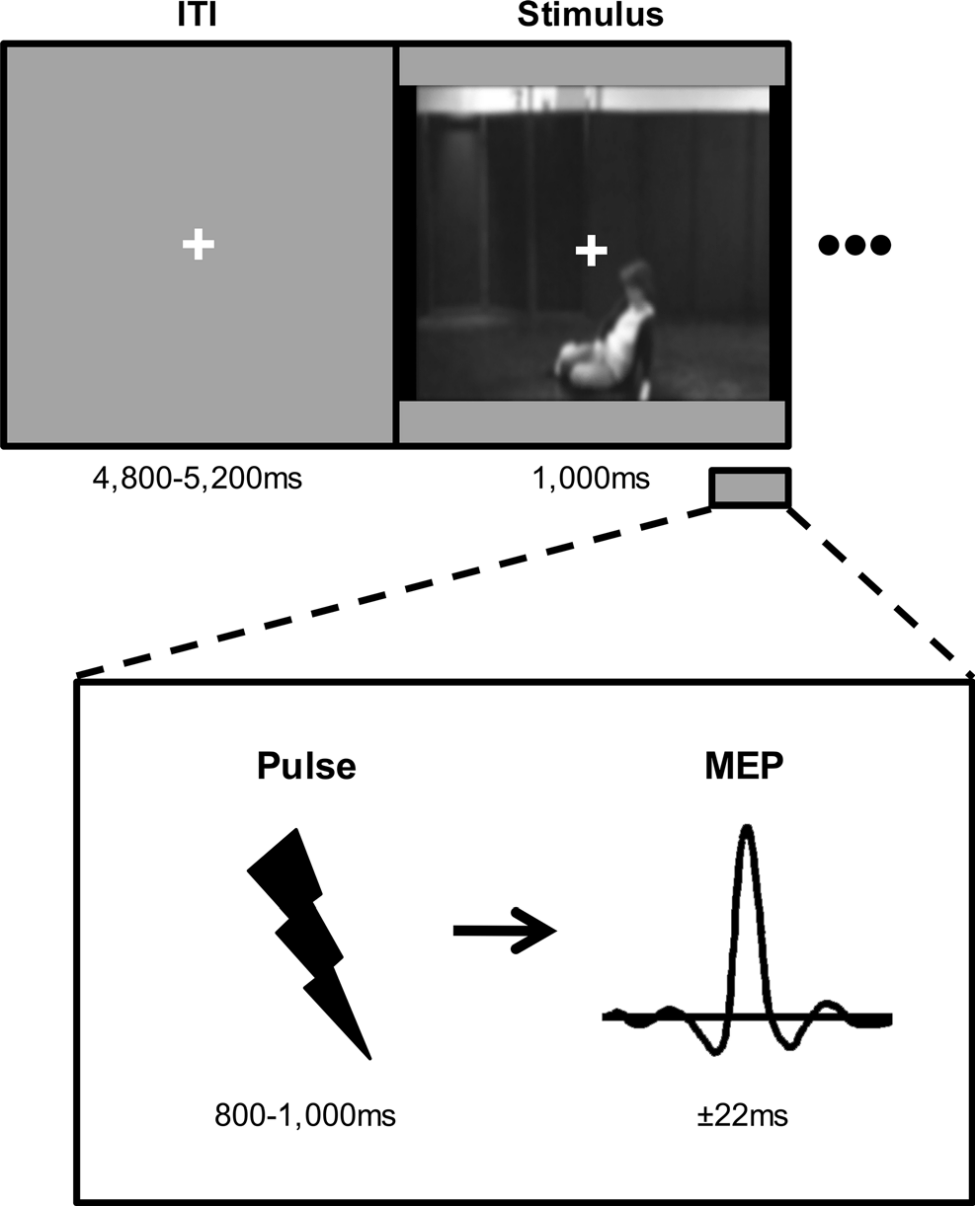
Transcranial magnetic stimulation (TMS) task. Motor-evoked potentials (MEPs) were recorded to a TMS pulse that was pseudorandomly delivered between 800 and 1,000 ms after video clip onset. ITI, intertrial interval

Upon arrival at the laboratory, the experimenter explained the procedure, and participants provided written informed consent and answered several questions on their current physical and mental well-being. Participants were seated in a comfortable dentist chair, with their arms placed on the upper leg and the palm of the hand facing upward. The resting motor threshold (MT) of the left hemisphere was assessed by means of the standardized visual thumb movement procedure (Schutter & van Honk, 2006). During the task, the TMS intensity was set at 120 % MT. Participants were instructed to relax their body and not to focus on their hands, but to fixate on the fixation cross shown continuously during the task. Participants did not need to respond during stimulus presentation. The same stimuli as in Experiment 2 were presented in a random order, with a blank screen with a fixation cross (4,800–5,200 ms) in between. Thus, six conditions were used, with the Situation (emergency vs. nonemergency) and the Number of Bystanders (no, one, or four bystanders) as within-subjects factors. As in the previous two experiments, no mention was made with respect to the content of the movies. The TMS pulse was pseudorandomly delivered between 800 and 1,000 ms (in six steps of 40 ms) after stimulus onset. This procedure is commonly used in single-pulse TMS studies to prevent anticipation by the participants (e.g., Avenanti et al. 2005). In the present study, the timing of the pulse did not affect MEP amplitudes, as was shown by a GLM for repeated measurements with Timing of Pulse (six levels) as a within-subjects factors, *F*(5, 100) = 0.82, *p =* .51. See Fig. 4 for the TMS procedure. As in Experiments 1 and 2, the procedure started with three practice trials (woman standing and waiting), followed by the scrambled videos of the scenarios serving as the baseline (12 trials), and finally, random presentation of the six scenarios (12 trials per condition). As in the previous two experiments, participants completed the IRI at the end of the experiment. Upon completion, participants were debriefed and received payment.

#### Data analysis

The data from one participant were removed because of a noisy and unstable electromyography (EMG) signal, and the data from a second participant were removed because of failure to comply with the instructions (i.e., excessive movement during testing). The MEP was quantified as the peak-to-peak amplitude of the maximal EMG response. Every trial was visually inspected, which was done blind to the stimulus condition. Trials containing excessive background EMG and abnormal MEPs were removed. The mean ± *SD* percentage of trials removed across participants was 4.37 ± 4.15 %. MEP amplitudes were calculated as the percentage change from the MEP amplitude during the baseline (scrambled) block.

For the statistical analyses, a procedure similar to that described in Experiment 2 was followed. First, to test the influence of the number of bystanders on MEP amplitudes during the observation of an emergency versus a nonemergency, a GLM for repeated measurements with Situation (two levels) and Number of Bystanders (three levels) as within-subjects factors was used.

Next, we calculated the slopes of the MEP amplitudes with the increase in the number of bystanders for the emergency and nonemergency situations for each individual. A negative slope would indicate that the MEP amplitude decreased as a function of the number of bystanders. A decrease in MEP amplitude would reflect a decrease in action preparation, and an increase in amplitude would reflect an increase in action preparation. To examine the relation between trait personal distress and the effect of the number of bystanders on MEP amplitudes, a linear regression analysis was employed similar to the one in Experiment 2. Trait personal distress was entered in the first step, and in Step 2 the three other trait empathy scores were added to the model in a stepwise fashion. The trait empathy scores of one participant were missing, resulting in a sample of 20 participants for the regression analyses.

### Results

TMS was well tolerated, and no adverse events occurred. Table 4 shows the MEP amplitudes across conditions. No main effect of situation, *F*(1, 20) = 0.97, *p =* .34, or number of bystanders, *F*(2, 40) = 0.54, *p =* .59, nor an interaction between situation and number of bystanders, *F*(2, 40) = 0.30, *p =* .74, was found. Additionally, we tested whether trait personal distress predicted the effect of the increase in the number of bystanders on motor corticospinal excitability levels during an emergency. A significant linear regression model was observed for the emergency slope, *F*(1, 17) = 5.42, *p* = .03, *R*^2^ = .24, ƒ^2^ = 0.32 (Table 5). In line with expectations, personal distress was negatively related to the effect of the number of bystanders on motor corticospinal excitability levels during an emergency, β = −.49, *p* = .03.^3^ No significant model emerged for the nonemergency slope, *F*(1, 19) = 0.77, *p =* .39, *R*^2^ = .04.

**Table 4:** Mean motor-evoked potential amplitudes ± standard errors, as percentage changes from baseline for Experiment 3

	No Bystanders	One Bystander	Four Bystanders
Emergency	133.94 ± 14.06	142.39 ± 14.72	140.18 ± 14.33
Nonemergency	133.32 ± 11.68	133.35 ± 14.55	134.74 ± 14.47

**Table 5:** Outcome of the regression analysis of the emergency slopes in Experiment 3

	*b*	β	*p*
*Step 1*			
Overall model: *F*(1, 17) = 5.42, *p* = .03, *R* ^2^ = .24, ƒ^2^ = 0.32			
Constant	14.22 ± 5.69 [2.22, 26.22]		.02
Personal distress	−1.32 ± 0.57 [−2.52, −0.12]	–.49	.03
*Step 2*			
Sympathy^*^		–.15	.54
Perspective taking^*^		.18	.42
Fantasy^*^		.25	.26

*b* = unstandardized coefficients ± standard errors [95 % confidence intervals], β = standardized coefficient. ^*^Removed predictors

### Discussion

In the third experiment, we examined the effect of the number of bystanders during an emergency situation on a direct measure of the motor system by using single-pulse TMS. No linear decrease in motor corticospinal excitability levels was observed with an increasing number of bystanders when participants witnessed an emergency. In line with our expectations, people with higher levels of personal distress showed a stronger decrease in motor corticospinal excitability levels during the observation of an emergency when the number of bystanders increased.

In the previous two experiments, we observed that both personal distress and perspective taking were associated with the effect of the number of bystanders on responding to an emergency. Using a direct measure of the physiological state of the motor system, we found that only personal distress, and not sympathy or perspective taking, predicted the effect of the number of bystanders. These results suggest that the effect of bystanders on the initial response to an emergency may indeed be related to action preparation (Hortensius & de Gelder, 2014). To further quantify this relation between personal distress and the effect of bystanders, and to disentangle the influences of perspective taking and personal distress on action preparation, we studied the influences of automaticity and cognitive involvement on these processes in a final experiment.

Several studies have started to explore whether reactions to distressful events are automatic (e.g., Gu & Han, 2007; Morelli & Lieberman, 2013; Rameson, Morelli, & Lieberman, 2012; Yamada & Decety, 2009). For example, Rand and Epstein (2014) showed that the decision-making process for extreme altruistic acts can be described as fast, intuitive, and reflexive. Of course, it is not an all-or-nothing mechanism. Some aspects of an empathic reaction can be automatic and reflexive, while others are deliberate and reflective in nature. Fan and Han (2008) showed that late, but not early, components are influenced by task manipulations. Moreover, interindividual differences in terms of automaticity and the attentional malleability of empathic responses have been reported (Rameson et al., 2012). Individuals with higher levels of trait empathy showed no reduction in empathic responses when performing an unrelated task, suggesting a more automatic process underlying these responses in these individuals. However, this study did not look at different aspects of empathy.

Although sympathy and personal distress are both considered to be part of a larger affective empathy cluster, they differ in terms of cognitive involvement. Whereas conditioning, direct association, and simple labeling or categorization of the emergency can lead to personal distress, they do not lead to feelings of sympathy per se (Eisenberg, Shea, Carlo, & Knight, 2014). Personal distress also requires minimal cognitive processes (Eisenberg & Fabes, 1990; Eisenberg et al. 2014), whereas sympathy requires more elaborate or more complex cognitive processes. Similarly, perspective taking—the capacity to understand the thoughts and feelings of another individual (Davis, 1980)—requires more sophisticated, top-down processes (Davis, Conklin, Smith, & Luce, 1996). This dissociation between personal distress, on the one hand, and sympathy and perspective taking, on the other hand, is also reflected in relations with prefrontal functions (Spinella, 2005). Trait personal distress is related to more executive dysfunction, whereas perspective taking and sympathy are inversely related to executive dysfunction.

One possible way to disentangle the influences and needs for cognitive processes in personal distress, sympathy, and perspective taking, and the possible automaticity of these reactions, would be to use a cognitive load manipulation. By imposing a cognitive load during the occurrence of another task, the dynamics between cognitive processes and the behavior of interest can be established. Under low-load conditions, the cognitive system is accessible and can influence behavior. This behavior can be described as being reflective, deliberate, or explicit. Under high-load conditions, the cognitive system is engaged and relatively inaccessible. If a particular behavior occurs during a high-cognitive-load manipulation, it is indicative of an automatic or reflexive mechanism (Gilbert, Pelham, & Krull, 1988), since these processes are not dependent on cognition.

By using a cognitive load manipulation during the cued reaction time task, we aimed to extend the previous findings and to dissociate the influences of trait perspective taking and personal distress on the negative influence of bystanders. Given the foregoing, we hypothesized that under conditions of high cognitive load, only trait personal distress would predict the slowing of reaction times during an emergency with bystanders present. If trait perspective taking were related to the effect of bystanders, this would only be apparent under conditions of low cognitive load. In line with the previous findings, we expected that sympathy would not predict the effect of bystanders during the observation of an emergency under either low- or the high-cognitive-load conditions.

## Experiment 4

### Method

#### Participants

A group of 39 female and 11 male volunteers (43 right-handed, six left-handed, one ambidextrous), between 18 and 28 years of age, participated in exchange for course credits. The participants were naïve to the aim of the study and provided informed consent. The experiment was carried out in accordance with the standards set by the Declaration of Helsinki.

#### Task and procedure

The cued reaction time task was slightly adapted to allow a cognitive load manipulation. Participants were instructed to remember a single two-digit number (e.g., 12; low cognitive load) or a combination of three two-digit numbers (e.g., 24, 36, 87; high cognitive load) while performing the cued reaction time task. Before each block of the task, a screen with a load instruction was presented to the participant for 2,500 ms. This was followed by a block of eight reaction time trials with 25 % no-go trials. After each block a memory probe was shown, and participants were instructed to indicate which of the two numbers had been part of the original sequence.

At the start of the experimental session, participants first practiced the cued reaction time task in isolation (three trials), followed by two practice blocks with the cognitive load manipulation (one low- and one high-cognitive-load block, each with three reaction time trials). Participants were instructed to remember the number presented at the start of each block while simultaneously performing the task. For the practice trials, video clips of a woman standing and waiting were used, whereas for the main experimental blocks, only the emergency and nonemergency scenarios with no and four bystanders were used. In the first half of the experiment, only the emergency and nonemergency situations with no bystanders were shown, followed by the emergency and nonemergency situations with bystanders present. This was done in order to measure the initial response to an emergency first without bystanders, and subsequently to assess the impact of bystanders on the response to an emergency, without trial-to-trial fluctuations and across-trial influence (cf. Rameson et al., 2012). The cognitive load manipulation was presented in a randomized order throughout the experiment, whereas the emergency and nonemergency situations were presented in a randomized order within each block. Participants performed 32 blocks in total, resulting in 24 go trials per condition. After the cued reaction time task, participants completed the Dutch version of the IRI.

#### Data analysis

The cognitive load manipulation was successful, so that accuracy decreased in the high-cognitive-load condition (mean ± *SD* percentage correct: 86.50 ± 11.11 %) as compared to the low-cognitive-load condition (92.13 ± 11.97 %), *t*(49) = 3.41, *p* = .001, *d* = 0.48. Filtering of the reaction times was performed as in Experiments 1 and 2 (mean ± *SD* percentage of trials removed, 0.63 ± 0.96 %; mean ± *SD* percentage of false alarms and misses, 2.94 ± 2.26 %). To calculate a bias score, we subtracted the reaction times for the situations with bystanders present from the reaction times for situations with no bystanders present, individually for the emergency and nonemergency situations in both the low- and high-cognitive-load conditions. Negative values indicated slower responses to the situation with bystanders present, and thus a stronger bystander effect, whereas positive values indicated faster responses to the situations with bystanders present. Next, we corrected for general task effects by performing a regression that predicted the bias scores in each of the four conditions based on the task effect (accuracy low – high cognitive load). By using the standardized residual of each of the bias scores, the variance explained by the overall task performance was removed, and the unique contributions of each condition could be examined. Next, linear regression analyses were used to predict the bias scores for the emergency and nonemergency situations under conditions of low and high cognitive load. As with the previous experiments, in the first step the hypothesized predictors were entered into the model (with low cognitive load, trait perspective taking; with high cognitive load, trait personal distress), and the remaining scales were added in Step 2 (in a stepwise fashion).

### Results

**Fig. 5:**
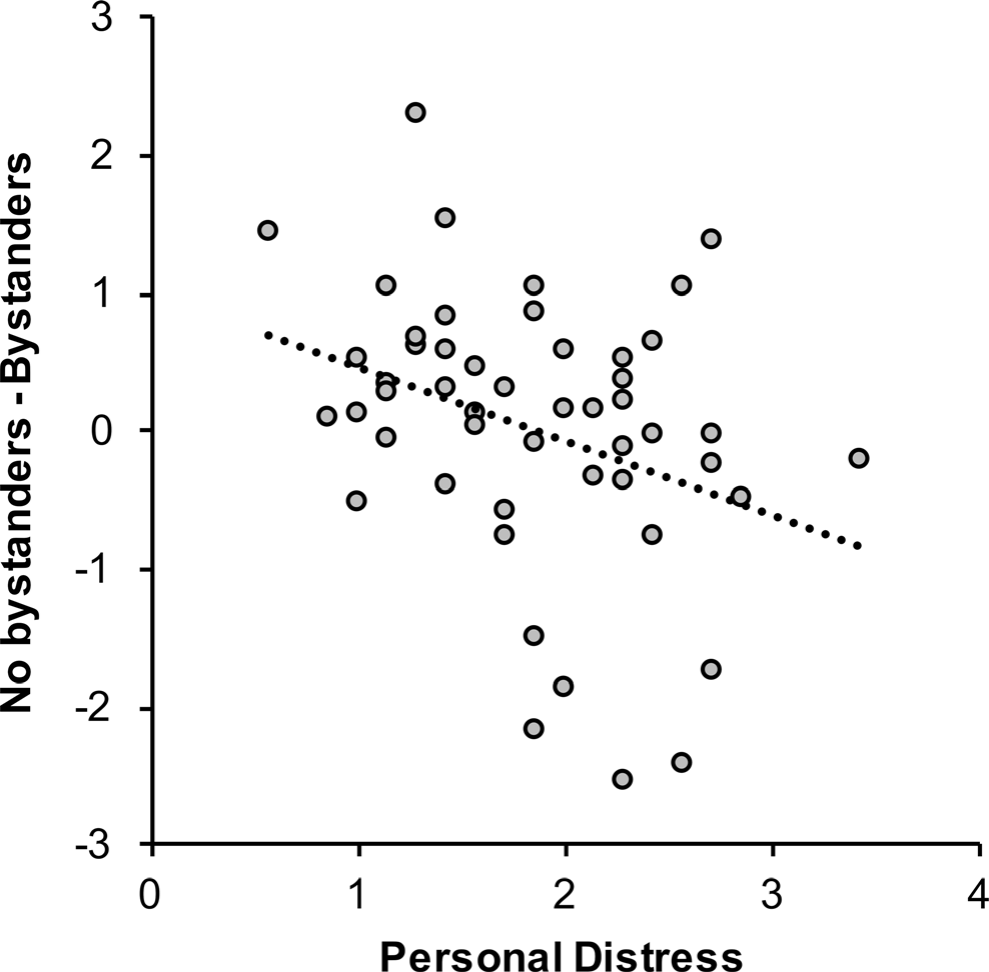
Under high cognitive load, trait personal distress predicted the slowing of responses when bystanders were present during an emergency

In the low-cognitive-load condition, no significant linear regression model was found for the emergency, *F*(1, 48) = 0.98, *p =* .33, *R*^2^ = .02, or the nonemergency, *F*(2, 47) = 2.58, *p =* .09, *R*^2^ = .10, situation. In the high-cognitive-load condition, a significant linear regression model was observed for the emergency situation, *F*(1, 48) = 6.02, *p* = .02, *R*^2^ = .11, ƒ^2^ = 0.12 (Table 6), but not for the nonemergency situation, *F*(1, 48) = 1.53, *p =* .22, *R*^2^ = .03. Crucially, trait personal distress predicted a stronger slowing of responses to an emergency situation with bystanders than with no bystanders present, β = −.33, *p* = .02 (Fig. 5). Neither sympathy nor perspective taking predicted the effect of bystanders: β = −.12, *p* = .44, and β = .09, *p* = .52, respectively.

**Table 6:** Outcome of the regression analysis for the no bystander – bystander bias scores in the emergency situation with high cognitive load in Experiment 4

	*B*	β	*p*
*Step 1*			
Overall model: *F*(1, 48) = 6.02, *p* = .02, *R* ^2^ = .11, ƒ^2^ = 0.12			
Constant	0.99 ± 0.42 [0.14, 1.84]		.02
Personal distress	−0.53 ± 0.22 [−0.97, −0.10]	–.33	.02
*Step 2*			
Sympathy^*^		–.12	.44
Perspective taking^*^		.09	.52
Fantasy^*^		–.22	.14

*b* = unstandardized coefficients ± standard errors [95 % confidence intervals], β = standardized coefficient. ^*^Removed predictors.

### Discussion

In our final experiment, we used the cued reaction time task combined with a cognitive load manipulation to influence cognitive involvement during the observation of an emergency with bystanders present. In agreement with our expectations, the results showed that personal distress predicted the slowing of responses to an emergency with bystanders present during the high-cognitive-load condition. In other words, people with higher levels of personal distress demonstrated stronger response slowing to an emergency with bystanders present when cognitive involvement was restricted. Under conditions of low cognitive load, and thus without cognitive restriction, neither personal distress nor perspective taking predicted an effect of bystanders on responding to an emergency. Sympathy was not associated with an effect of bystanders on responses to an emergency in either the presence or the absence of cognitive restriction. The results of Experiment 3 indicated that personal distress is predictive of a mechanism related to action preparation, and the results of Experiment 4 extended these findings in an important manner. Personal distress predicted an effect of bystanders on an initial response to an emergency that was more related to automatic, reflexive action preparation.

## General discussion

The aim of the present study was to investigate the influence of bystanders on the responses of individuals to an emergency situation by integrating situational and dispositional factors. In a series of four experiments, we examined the differential effects of trait sympathy and personal distress on the willingness to help with bystanders present. The results showed that even though personal distress and sympathy predicted overall faster responses to an emergency when no bystanders were present, personal distress was most consistently predictive of a decrease in action preparation when bystanders were present during an emergency. These results are in line with findings showing differences between other-oriented and self-centered responses to emergency situations in terms of sensitivity to social context. Our observations show that the effect of bystanders is already present at the level of action preparation. This bystander effect is proposed to be stronger for people with a predisposition to experience self-centered empathic responses, as measured by trait personal distress.

Our findings add to the growing body of evidence on how empathic responses are modulated by situational and dispositional factors (Decety & Lamm, 2009), as well as how sympathy and personal distress differ in sensitivity to social context (Archer et al. 1981; Batson et al., 1981; Batson et al., 1987; Batson et al., 1983; Carlo et al., 1991; Coke et al. 1978; Eisenberg, Fabes, et al., 1989; Fultz et al. 1986; Romer et al., 1986; Toi & Batson, 1982). Decety and Jackson (2004) argued that three interrelated mechanisms underlie the variety of empathic responses; perception–action coupling (see also Preston & de Waal, 2002), emotion regulation mechanisms, and perspective taking. Since trait personal distress as well as sympathy measure affective responses to the distress of others (Davis, 1983), one possibility is that a disposition to experience and regulate negative emotions underlies this difference in sensitivity to social context. Studies have shown a positive relation between a disposition to experience personal distress and heightened behavioral and physiological responses to social–emotional situations and decreased regulation of these responses (Avenanti et al. 2009; Borgomaneri et al. 2014; Eisenberg & Fabes, 1990; Eisenberg et al. 1994; Ferri et al. 2010; Okun, Shepard, & Eisenberg, 2000). In contrast, trait levels of sympathy have been linked to increased emotion regulation (Eisenberg et al. 1996; Okun et al., 2000). Using single-pulse TMS, we showed that personal distress but not sympathy predicts the negative influence of bystanders on motor corticospinal excitability levels as indexed by MEPs. In line with previous studies (Avenanti et al. 2009; Borgomaneri et al. 2014; Ferri et al. 2010), these results suggest that a distinction between sympathy and personal distress can be observed already in the action domain. A disposition to experience personal distress in contrast to sympathy thus can not only lead to an imbalance in higher, regulatory-related processes, but may already have an influence on a lower, action-related processes. Although the default mode is to help—that is, intact coupling between situation and response—the presence of bystanders may result in a decoupling. This effect may be stronger in people with higher levels of personal distress, who display attenuated action preparation to respond to the emergency situation in the presence of bystanders.

What could drive this decoupling? The perception–action arc is motivation-dependent (Carver, 2006; Mogenson, Jones, & Yim, 1980). The state of the motor cortex (Schutter, de Weijer, Meuwese, Morgan, & van Honk, 2008; Schutter, Hofman, Hoppenbrouwers, & Kenemans, 2011) and the multifaceted concept of empathy (Gutsell & Inzlicht, 2012; Tullett, Harmon-Jones, & Inzlicht, 2012; Zaki, 2014) have been linked to both approach- and avoidance-related motivation. As was described by Preston (2013), the distinction between avoidance and approach is crucial in explaining the lack of helping behavior in some situations. For onlookers to respond to an emergency, the event has to be classified as a threat, which consequently triggers either approach- or avoidance-related behavior. Several explicit or implicit strategies can result in the approach toward or avoidance of current or future empathic responses (Zaki, 2014). For example, the avoidance can be overt (need to escape the situation) or covert (attentional disengagement). Graziano and Tobin (2009) described the approach–avoidance dimension of an emergency situation. They suggested that two evolutionarily conserved motivational systems, fight-or-flight, which includes freezing behavior (the fight–freeze–flight system; e.g., McNaughton & Corr, 2004), and parental care, are activated when we encounter a novel or distressful event, and these two systems act as opponents to each other’s dominant action patterns. Incorporating the opponent-process model of motivation by Solomon and colleagues (Solomon, 1980; Solomon & Corbit, 1974), the authors stated that the first, and fastest, response to an emergency is that of distress (Process A, fight–freeze–flight system), and if the possibility to escape the situation is available and easy, helping behavior does not occur. However, over time the slower reaction of sympathy (Process B, care system) is activated, opposing the fixed action patterns of personal distress (Graziano & Habashi, 2010; Graziano & Tobin, 2009). This nicely fits the observation in the first experiment that, although sympathy and personal distress are opposing constructs, they both predicted faster responses to an emergency without bystanders present, and also the notion in the literature that they are positively correlated and can exist in parallel within an individual (Batson et al., 1987; Davis, 1980). However, the presence of bystanders during an emergency possibly increases Process A (distress, the fight–freeze–flight system), leading to heightened distress and mitigating preparation of helping behavior, whereas Process B (sympathy, care system) is not affected by the presence of bystanders. This bystander-mediated increase in distress in the onlooker and increased activation of the fight–freeze–flight system is only apparent in people with a disposition to experience personal distress. However, it remains unknown whether and how this increase in state levels of distress occurs and what the dynamics of a personal distress state–trait interaction would be.

It is possible that the observed effect might not be related to a decrease in action preparation or inhibition of approach, but rather to a freeze-like response. This amounts to a reduction in motor corticospinal excitability with an increase in the number of bystanders during an emergency. Thus, this reduction might reflect increased freezing in people with a disposition to experience personal distress. Freezing occurs when there is no possibility to escape the situation (or predator), or as an initial phase in a response (McNaughton & Corr, 2004). Several arguments complicate the interpretation of the decrease in motor corticospinal excitability as a freezing motor plan. First, a freeze-like reduction in motor corticospinal excitability has been recorded 100–125 ms post-stimulus-onset (Borgomaneri, Vitale, Gazzola, & Avenanti, 2015). In the present study, we stimulated in a time window from 800 to 1,000 ms post-stimulus-onset, making it unlikely that we tapped into a freeze-like motor program. Second, both state and trait levels of personal distress are related to enhanced motor corticospinal excitability to stimuli that are negative in valence, which is contrary to a freeze-related reduction in excitability levels (Avenanti et al., 2009; Borgomaneri et al. 2014). Third, we measured MEPs from an extensor muscle, the APB. Although the link between extensor and flexor muscles and approach and avoidance motivation is complicated (Krieglmeyer & Deutsch, 2013; Phaf, Mohr, Rotteveel, & Wicherts, 2014), recordings of APB and other muscles have been linked to approach motivation (Coombes et al., 2009; Schutter, Hofman, & van Honk, 2008). To shed more light on the issues of approach and avoidance motives, future studies may incorporate different TMS procedures that could allow the measurement of inhibitory processes, recordings from multiple muscle groups at several time periods, and more direct measures of freezing, motivation, and prosocial behavior, to disentangle these different processes. In addition, future research could use different situations (e.g., a person directly being threatened by another individual with bystanders present) to shed light on the motives in the onlooker.

Helping behavior is thought to be driven by an evolutionarily conserved mechanism, reflexive in nature (Preston, 2013) and shared with other species (Preston & de Waal, 2002), that is the end result of both bottom-up and top-down processes. This is not to say that the decoupling is a deliberate, cognition-driven process. In the final experiment, personal distress predicted the effect of bystanders on responding to an emergency under conditions of high cognitive load. This suggests that the bystander effect is apparent not only at an explicit, cognitive level, but also at an implicit, automatic, action-related one. The perception–reaction arc can be automatic but still be context-dependent (Gawronski & Cesario, 2013). Although some research has suggested that the perception of the need of others is not automatic (e.g., Gu & Han, 2007; Rameson et al., 2012), it is important to note that most studies have focused on the perception of the need and distress of others. In the present study, we highlighted the reactive aspect by measuring reaction times and motor corticospinal excitability to the distress of others. In contrast with other studies that have tended to be biased toward explicit cognitive processes (intention or mental states), we focused on perception–action coupling as a function of context.

In the second experiment, we observed that perspective taking and not personal distress predicted the negative effect of bystanders on responding to an emergency. What is the role of perspective taking? How does this relate to the effect of personal distress and the two-system perspective? Multiple mechanisms can exist in parallel to influence responses to, and helping behavior during, an emergency situation with bystanders. The prosocial individual is the combined sum of situation-dependent cognition (e,g, perspective taking), feelings (e.g., personal distress or sympathy), and behavior (Habashi & Graziano, 2015). Of course, these components interact, and perspective taking can lead to an increase in state sympathy (Batson et al. 1989; Batson et al. 1988; Toi & Batson, 1982). For example, instructing participants to take the perspective of rather than simply to observe the victim increases sympathy, whereas personal distress remains unchanged (Toi & Batson, 1982). These interactions are also found for trait levels: Perspective taking is positively correlated with sympathy, but inversely correlated with personal distress (Davis, 1980; De Corte et al., 2007). One hypothesis that partly reconciles the results is that perspective taking, by means of its interaction with sympathy, is related to Process B (sympathy and the care system). Although Process B is not influenced directly by the presence of bystanders, it is influenced indirectly, by means of perspective taking, which thus sustains a form of cognitive influence on Process B. Together, situational influences on both Processes A and B might be mediated by trait levels of personal distress and perspective taking, respectively. How the slowing of responses, as predicted by perspective taking, is related to helping behavior remains to be investigated.

The majority of participants were female college students, and the question arises whether the present findings can be generalized to the population at large. Popular belief holds that women score higher on empathy-related constructs, but that men are more likely to provide help. So far, no consistent sex differences in helping behavior have been reported (Eagly & Crowley, 1986). In a recent meta-analysis based on all studies of the bystander effect between 1960 and 2010, no significant sex difference was found (Fischer et al., 2011). Importantly, Tice and Baumeister (1985) showed that sex or the level of femininity did not influence the occurrence of helping behavior when bystanders were present. Only participants high in masculinity provided less help. It has been argued that sex differences in helping behavior (and other empathy-related processes) are all about gender roles (Eagly & Crowley, 1986; Senneker & Hendrick, 1983). These differences only emerge when gender roles are primed by means of the social context, such as by demand characteristics or the type of helping behavior studied, and are motivation-dependent (Eisenberg & Lennon, 1983; Ickes, Gesn, & Graham, 2000; Klein & Hodges, 2001). Although sex differences or the impact of gender roles on the processing of an emergency and helping behavior were not part of our primary research question, they may be of interest for future research.

Another issue with both methodological and theoretical consequences that could be addressed in future research is the effect of repetition on responses to emergency situations with or without bystanders. Of course, a certain number of trials will be necessary to achieve a reliable measure of reaction times, but it might also have several important consequences. Reaction times might change as a function of repetition number. Although effects of repetition on stimulus–response relationships have been studied (Bertelson, 1965; Felfoldy, 1974), the interactions with stimulus condition and personality characteristics remain unknown. How do multiple repetitions of an emergency situation interact with a disposition to experience personal distress? On the basis of application of the model described by Graziano and Tobin (2009) to the present data, repetition could have differential effects on Process A (distress and the fight–freeze–flight system) and Process B (sympathy and the care system). One hypothesis conceptually driven by the work of Solomon and colleagues (Solomon, 1980; Solomon & Corbit, 1974) suggests that with increased repetition or exposure to emergencies, the dominance of Process A diminishes, whereas Process B increases in strength (Graziano & Habashi, 2010). This would then result in a decrease in distress-driven processes and an increase in sympathy-driven processes. In the present context, one would thus expect that with an increase in repetition, personal distress would eventually not be predictive of the negative effect of bystanders on responding to an emergency. However, repetition effects can take multiple forms and shapes (Grill-Spector, Henson, & Martin, 2006), and the interactions with stimulus condition and dispositional factors need to be investigated in carefully designed experiments.

In conclusion, using a person–situation approach, we have extended the existing literature on the psychological and neural bases of the bystander effect by showing that the presence of bystanders during an emergency attenuates action preparation for people with higher levels of trait personal distress—that is, with a disposition to experience self-centered empathic responses.

## Electronic supplementary material

Below is the link to the electronic supplementary material.

ESM 1 (MOV 1.04 mb)

## Footnotes

1 One individual with a slope (−5.21) three standard deviations from the mean in the emergency situation was removed from the analyses. With inclusion of this individual, the slopes for the emergency (0.29 ± 0.28) and nonemergency (0.11 ± 0.25) situations were not significantly different from each other, *t*(29) = 0.63, *p* = .54, or from zero, *t*(29) = 1.06, *p* = .30, and *t*(29) = 0.44, *p* = .66, respectively.

2 With inclusion of the outlier, a significant model appeared, *F*(2, 27) = 5.11, *p =* .01, *R*^2^ = .28, ƒ^2^ = 0.39, in which personal distress, β = .38, *p* = .03, and perspective taking, β = .42, *p* = .02, predicted the emergency slope.

3 As in Experiments 1 and 2, we checked for outliers. In line with Cook and Weisberg (1982), we removed one individual with a Cook’s distance of 1.39. With this individual included, a similar, but weaker, model emerged, *F*(1, 18) = 3.72, *p =* .07, *R*^2^ = .17, ƒ^2^ = 0.20, in which personal distress predicted the emergency slope, β = −.41, *p* = .07.
